# Cell type-specific anti-cancer properties of valproic acid: independent effects on HDAC activity and Erk1/2 phosphorylation

**DOI:** 10.1186/1471-2407-10-383

**Published:** 2010-07-21

**Authors:** Kamil Gotfryd, Galina Skladchikova, Eugene A Lepekhin, Vladimir Berezin, Elisabeth Bock, Peter S Walmod

**Affiliations:** 1 Protein Laboratory, Department of Neuroscience and Pharmacology, Faculty of Health Sciences, University of Copenhagen, Denmark

## Abstract

**Background:**

The anti-epileptic drug valproic acid (VPA) has attracted attention as an anti-cancer agent.

**Methods:**

The present study investigated effects of VPA exposure on histone deacetylase (HDAC) inhibition, cell growth, cell speed, and the degree of Erk1/2 phosphorylation in 10 cell lines (BT4C, BT4Cn, U87MG, N2a, PC12-E2, CSML0, CSML100, HeLa, L929, Swiss 3T3).

**Results:**

VPA induced significant histone deacetylase (HDAC) inhibition in most of the cell lines, but the degree of inhibition was highly cell type-specific. Moreover, cell growth, motility and the degree of Erk1/2 phosphorylation were inhibited, activated, or unaffected by VPA in a cell type-specific manner. Importantly, no relationship was found between the effects of VPA on HDAC inhibition and changes in the degree of Erk1/2 phosphorylation, cell growth, or motility. In contrast, VPA-induced modulation of the MAPK pathway downstream of Ras but upstream of MEK (i.e., at the level of Raf) was important for changes in cell speed.

**Conclusions:**

These results suggest that VPA can modulate the degree of Erk1/2 phosphorylation in a manner unrelated to HDAC inhibition and emphasize that changes in the degree of Erk1/2 phosphorylation are also important for the anti-cancer properties of VPA.

## Background

Valproic acid (2-n-propylpentanoic acid, VPA) is a common anti-epileptic drug that is also used for the treatment of bipolar disorder, migraine and neuropathic pain [1]. Moreover, VPA can modulate several cancer-related processes, including angiogenesis, immunogenicity, and invasion, metastasis, differentiation, proliferation and apoptosis of cancer cells [2]. Recent clinical studies have demonstrated the chemotherapeutic efficacy of VPA for the treatment of several types of cancer, including acute myeloid leukemia, myelodysplastic syndromes [3], and solid breast and cervix tumors [4].

VPA is a histone deacetylase (HDAC) inhibitor that alters gene expression, thereby modulating processes such as cell growth, differentiation and apoptosis [5,6]. The drug is also known to modulate the activity of several intracellular enzymes, including mitogen-activated protein kinases (MAPKs), protein kinase C (PKC) and glycogen synthase kinase-3β (GSK-3β) [2,7].

Several of the cellular processes modulated by VPA may be partially regulated by signaling through the MAPK pathway [8-10]. Signaling through this pathway is often initiated by activation of membrane-localized receptors, leading to activation of the GTPase Ras, and subsequent activation of the MAPK kinase kinase Raf, the MAPK kinases MEK1/2 and the MAPKs Erk1/2 [11]. Activated Erk1/2 phosphorylate targets in the cytosol and transcription factors in the nucleus [12]. Erk1/2 can also be activated Ras-independently by other upstream molecules, including protein kinase A (PKA) and PKC. Furthermore, cells express up to three Raf types (i.e., A-, B- and c-Raf), which are affected differently by upstream targets, thereby adding a further level of complexity to MAPK-mediated signaling [13,14].

The anti-cancer effects of VPA are generally attributed to its HDAC inhibitory activity. However, these effects may also be partially caused by alterations in, for example, Erk1/2 activity. Interestingly, previous studies have shown cell type-specific effects of VPA, both on specific cellular processes, such as cell migration and proliferation [15], and on specific enzyme activities, including Erk1/2 activity [16,17]. The reasons for these cell type-specific effects, however, are unknown.

The aim of the present study was to investigate the relationships between VPA-induced changes in HDAC and Erk1/2 activities, and cell growth and motility. The results reveal striking cell type-specific differences in the responses to VPA. Moreover, the effects of VPA on cell growth, motility and the degree of Erk1/2 phosphorylation were not related to its effects on HDAC inhibition. In contrast, modulation of cell growth and motility was in some cell lines related to changes in the degree of Erk1/2 phosphorylation, which was regulated at the level of Raf.

## Methods

### Cell cultures

Cells were grown in a humidified atmosphere at 37°C, 5% CO_2_. The rat glioma cell lines BT4C and BT4Cn, the human glioma cell line U87MG (a gift from Dr. Nina Pedersen, Copenhagen University Hospital Rigshospitalet, Copenhagen, Denmark), the mouse neuroblastoma cell line N2a, the mouse adenocarcinoma cell lines CSML0 and CSML100, the human adenocarcinoma cell line HeLa, and the mouse fibroblastoid cell lines Swiss 3T3 and L929 were grown in Dulbecco's modified Eagle's medium (DMEM) supplemented with 10% (v/v) heat-inactivated fetal calf serum (FCS), 2 mM GlutaMAX, 100 U/ml penicillin, 100 μg/ml streptomycin and 2.5 μg/ml fungizone (all from Invitrogen, Taastrup, Denmark). Cells were dislodged with trypsin/EDTA in modified Puck's saline (Invitrogen).

The rat pheochromocytoma cell line PC12-E2 was grown in DMEM supplemented with 5% (v/v) FCS, 10% (v/v) horse serum, 2 mM GlutaMAX, 100 U/ml penicillin and 100 μg/ml streptomycin (Invitrogen). Cells were dislodged by tapping the culture flask.

### Cell transfection

Stable transfection of L929 with the pGV16 vector encoding constitutively active rat H-Ras (G12V; a gift from Prof. Berthe Willumsen, Department of Biology, Faculty of Science, University of Copenhagen, Copenhagen, Denmark) was obtained using Lipofectin (Invitrogen). After transfection, cells were grown in medium containing 0.75 mg/ml geneticin (Invitrogen) for 3 weeks. Six geneticin-resistant clones with high H-RasG12V expression were selected, propagated, pooled and used as stock for subsequent experiments. Ten randomly selected clones stably transfected with the empty pGV16 vector were selected, propagated, pooled and used as control cells.

Transient transfections of cells with the pGV16 vector encoding dominant-negative rat H-Ras (S17N; a gift from Dr. Berthe Willumsen), the pRK5 vector encoding constitutively active rat MEK2 (S222E/S226E; a gift from Dr. Klaus Seedorf, Hagedorn Research Institute, Gentofte, Denmark), or the corresponding empty vectors were performed using FuGene 6 (Roche Diagnostics, Mannheim, Germany) or Lipofectin 2000 (Invitrogen). Transient transfections were performed as co-transfections with low amounts of the pEGFP-N1 vector (Clontech, Saint-Germain-en-Laye, France). Consequently, transfected cells were identified from their expression of enhanced green fluorescent protein. Cells were replated for subsequent experiments ~24 h after transfection.

### VPA treatment

A stock solution of 3 M VPA (Sigma-Aldrich, Copenhagen, Denmark) was prepared in dimethylsulfoxide (DMSO), and all assays were performed in the presence of 0.1% (v/v) DMSO in the presence or absence of VPA.

### Immunoblotting

Cells were plated in 60 mm culture dishes (Nunc, Roskilde, Denmark) at a density of 0.25-0.75 × 10^6 ^cells/dish and grown in medium containing 0-3 mM VPA for up to 48 h. In experiments in which cells were exposed to a MEK inhibitor (PD98059; Cell Signaling, Danvers, MA), the compound was added to the cultures 1 h before cell lysis. Following incubation, cells were rinsed in ice-cold PBS and collected in radioimmunoprecipitation assay lysis buffer supplemented with Complete, EDTA-free Protease Inhibitor Cocktail (Roche Diagnostics) and Phosphatase Inhibitor Cocktail Set III (Calbiochem). Proteins were resolved by sodium dodecyl sulfate-polyacrylamide gel electrophoresis (SDS-PAGE) and transferred to Immobilon-P membranes (Millipore, Billerica, MA). The membranes were blocked in PBS containing 0.1% (v/v) Tween-20 and 5% (w/v) bovine serum albumin.

Following incubation with primary antibodies, all membranes were incubated with horseradish peroxidase-conjugated goat anti-rabbit antibody (Cell Signaling), visualized by chemiluminescence (SuperSignal West Dura Extended Duration Substrate, Pierce Biotechnology, Rockford, IL) using a GeneGnome (Syngene, Cambridge, UK), and quantified using the accompanying GenTools software.

For estimation of Erk1/2 phosphorylation, membranes were probed with anti-phospho-Erk1/2 (Thr202/Tyr204) antibody (Cell Signaling). After visualization, the membranes were stripped (30 min, 50°C) in 62.5 mM Tris-HCl, pH 6.8, 2% (w/v) SDS and 50 mM 1,4-dithio-L-threitol, blocked, and reprobed with anti-pan-Erk1/2 antibody (Cell Signaling). Ras expression was determined using anti-H-, K- and N-Ras antibodies (Cell Signaling). Histone acetylation was determined using anti-acetyl histone H3 (Lys9/Lys14) antibody (Cell Signaling). Ras and histone H3 acetylation immunoblots were stripped and reprobed with anti-actin antibody (Sigma-Aldrich).

### Cell growth

Cell growth was quantified from crystal violet stainings. 5 × 10^3 ^cells/well were plated in 96-well microwell plates (Nunc) and grown in medium containing 0-3 mM VPA for 48 h, with each treatment replicated in 6 wells/plate. Cells were fixed in 3.7% (v/v) formalin and 1% (v/v) methanol in PBS, and stained with 0.04% (w/v) crystal violet in 4% (v/v) ethanol. Finally, the cell-bound crystal violet was solubilized using 1% (w/v) SDS, and optical density was measured at 600 nm.

### Measurement of individual cell motility

6-15 × 10^3 ^cells/well were plated in 6-well culture plates (Nunc) and grown in medium containing 0-3 mM VPA. Time-lapse video-recordings were performed using a computer-assisted microscope workstation as previously described [18]. Simultaneous, sequential recordings from 8-20 microscopic fields/well were performed at 1-10 min intervals for 20-80 min. Evaluation of individual cell motility was performed using the image processing software PRIMA (Protein Laboratory, Copenhagen University, Denmark). 50-200 cells from each well were tracked, and the data were used for the calculation of the mean-cell speed and the mean-squared cell displacement (<*d^2^*>) as previously described [19].

### Statistics

Results are expressed as mean ± SEM, calculated on the basis of the number of experiments. Unless indicated otherwise, statistical analyses were performed on non-normalized data using one-way repeated-measures analysis of variance (ANOVA) followed by the Tukey-Kramer multiple comparison test. Immunoblots were evaluated using the Wilcoxon signed rank test. Estimates of *IC*_*25 *_and *IC*_*50 *_values were based on the interpolation of data from log dose-response curves. Correlations were performed as Pearson correlations. All p-values refer to two-tailed calculations. *, ** and *** indicate p < 0.05, 0.01 and 0.001, respectively.

## Results

### Effects of VPA on the degree of Erk1/2 phosphorylation and histone H3 acetylation

**Figure 1:**
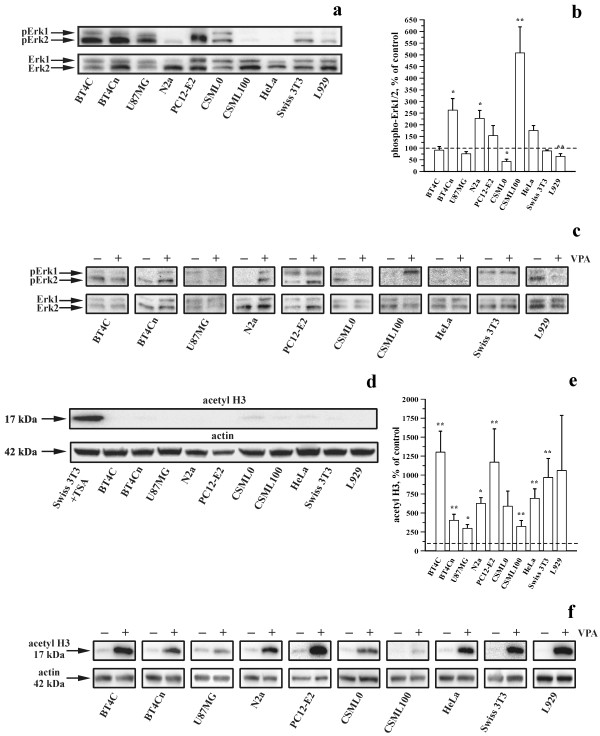
**Effects of VPA on the degree of Erk1/2 phosphorylation and histone H3 acetylation**. (a, d) Representative immunoblots showingdegree of Erk1/2 phosphorylation (a) and histone H3 acetylation (d) in cells grown in the absence of VPA for 48 h. (c, f) Representative immunoblots showing changes in the degree of Erk1/2 phosphorylation (c) and histone H3 acetylation (f) in cells grown in the absence or presence of 3 mM VPA for 48 h. The corresponding levels of Erk1/2 (a, c) or actin (d, f) are shown as loading controls. Protein levels are not comparable between different cell lines. As a positive control for histone H3 acetylation, Swiss 3T3 cells were treated with trichostatin A (TSA; 400 nM, 24 h) (d, first lane). (b, e) Bar graphs showing the effects of VPA (3 mM, 48 h) on the degree of Erk1/2 phosphorylation (b) and histone H3 acetylation (e). The degree of Erk1/2 phosphorylation and histone H3 acetylation were normalized to the corresponding cells untreated with VPA from 6-12 individual experiments.

Erk1/2 activity was investigated in 10 cell lines, including BT4C, BT4Cn, U87MG, N2a, PC12-E2, CSML0, CSML100, HeLa, Swiss 3T3 and L929, by estimating the degree of Erk1/2 phosphorylation by immunoblotting. In the absence of VPA, BT4C, BT4Cn, U87MG, and PC12-E2 exhibited high Erk1/2 phosphorylation levels. L929, Swiss 3T3 and CSML0 exhibited intermediate levels, and N2a, CSML100 and HeLa exhibited low levels (**Figure **1a). **Figure **1b and 1c show the relative changes in the degree of Erk1/2 phosphorylation in the respective cell lines in response to VPA (3 mM, 48 h). Erk1/2 phosphorylation was significantly decreased in CSML0 and L929, significantly increased in CSML100, BT4Cn and N2a, and not significantly affected in BT4C, U87MG, PC12-E2, HeLa and Swiss 3T3.

VPA is a known HDAC inhibitor. **Figure **1d shows the degree of acetylation of histone H3 in the 10 cell lines under control conditions determined by immunoblotting. All cell lines exhibited low acetylation levels under control conditions (**Figure **1d).

VPA significantly increased the degree of acetylation in 8 of the 10 cell lines (**Figure **1e and 1f). The changes in acetylation ranged from an approximately three-fold increase (U87MG) to an ~13-fold increase (BT4C; **Figure **1e).

In conclusion, VPA has highly cell type-specific effects on the degree of HDAC inhibition and on changes in the degree of Erk1/2 phosphorylation. Furthermore, the effects of VPA on the degree of Erk1/2 phosphorylation and HDAC inhibition were not related to the degree of Erk1/2 phosphorylation and acetylation in the absence of the drug. Finally, no relationship was found between the effects of VPA on HDAC inhibition and the degree of Erk1/2 phosphorylation. Thus, VPA-induced changes in the degree of Erk1/2 phosphorylation cannot be explained by the effects of the drug on HDAC activity, although HDAC inhibition may contribute to the observed effects.

### Effects of VPA on cell growth

**Figure 2:**
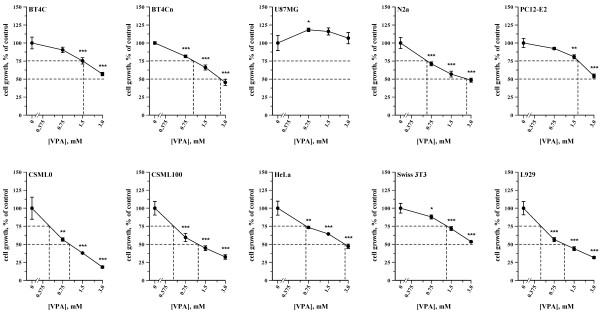
**Effects of VPA on cell growth**. Dose-response curves showing the effects of VPA on cell growth. Cells were exposed to VPA (0-3 mM, 48 h) before growth was determined using crystal violet staining. Results were normalized to the corresponding cells untreated with VPA from four independent experiments.

**Figure **2 shows that VPA dose-dependently changed the growth of all investigated cell lines. Nine cell lines exhibited IC_25 _values ranging from 0.43 to 1.83 mM (**Table **1), whereas the growth of a single cell line, U87MG, was significantly elevated upon exposure to 0.75 mM VPA.

**Table 1:** IC_25 _and IC_50 _values for the effects of VPA on cell growth.

Cell Line	***IC***_***25***_^**a **^**(mM)**		***IC***_***50***_^**a **^**(mM)**	
BT4C	1.52	(1.12 - 1.84)	>3.0	-
BT4Cn	1.06	(0.95 - 1.20)	2.67	(2.37 - 2.98)
U87MG	>3.0	-	>3.0	-
N2a	0.65	(0.54 - 0.72)	2.73	(2.11 - 3.19)
PC12-E2	1.83	(1.67 - 1.99)	>3.0	-
CSML0	0.43	(0.24 - 0.53)	1.01	(0.94 - 1.08)
CSML100	0.46	(0.32 - 0.58)	1.22	(0.98 - 1.41)
HeLa	0.70	(0.62 - 0.75)	2.75	(2.54 - 3.01)
Swiss 3T3	1.35	(1.22 - 1.47)	>3.0	-
L929	0.43	(0.32 - 0.51)	1.13	(0.97 - 1.29)

*IC*_*25 *_and *IC*_*50 *_values for the effects of VPA on cell growth calculated from interpolations of data. a: Numbers in parentheses indicate interpolated values for *IC*_*25 *_or *IC*_*50 *_± SEM (based on four independent experiments).

A correlation analysis of the data presented in **Figure **1 and 2 revealed no significant correlations between the effects of VPA on HDAC inhibition and cell growth or between the degree of Erk1/2 phosphorylation and cell growth (**Figure S4**). However, four of the five cell lines that did not demonstrate significant changes in the degree of Erk1/2 phosphorylation in response to VPA (**Figure **1b) did not have IC_50 _values for growth within the tested concentration range (below 3 mM; **Table **1), whereas all five cell lines demonstrating significant changes in the degree of Erk1/2 phosphorylation in response to VPA had IC_50 _values for growth below 3 mM. These results demonstrate a significant relationship between the effects of VPA o the degree of Erk1/2 phosphorylation and cell growth (p < 0.05, Fisher's exact test).

In conclusion, VPA induced highly cell type-specific effects on cell growth. Furthermore, no apparent relationship was observed between the effects of VPA on HDAC inhibition and cell growth, whereas VPA was likely to affect the growth of a given cell type if it affected the degree of Erk1/2 phosphorylation in that cell type at physiologically relevant concentrations.

### Effects of VPA on individual cell motility and the degree of Erk1/2 phosphorylation in L929 cells

Erk1/2 are known to regulate cell motility [20]. In L929 cells, VPA inhibits the degree of Erk1/2 phosphorylation (**Figure **1) and cell speed [21]. Therefore, a potential relationship between the effects of VPA on the degree of Erk1/2 phosphorylation and cell motility was investigated further in L929 cells.

**Figure 3:**
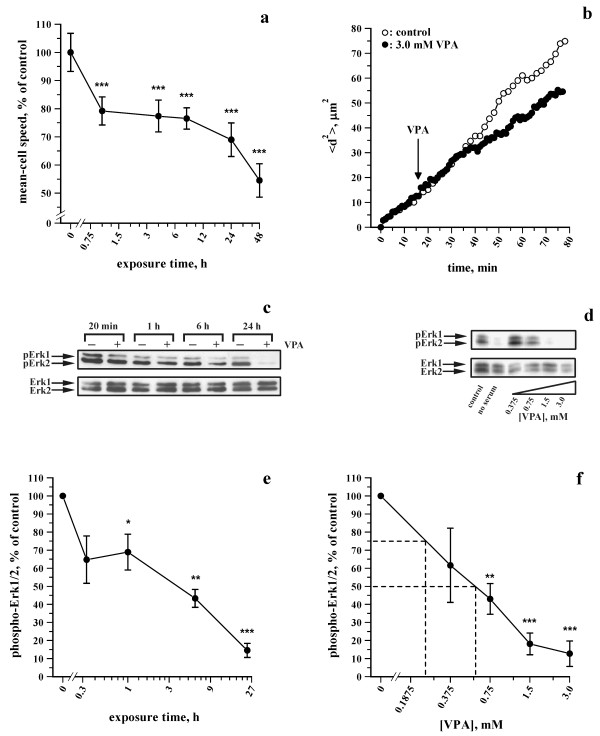
**Effects of VPA on individual cell motility and the degree of Erk1/2 phosphorylation in L929 cells**. (a) Time-response curve showing the effects VPA on mean-cell speed of L929 cells exposed to 3 mM VPA. Cells were recorded for 20 min at 2 min intervals. The mean-cell speed of the individual cells was normalized to the corresponding cells untreated with VPA from four to seven independent experiments. (b) Time-response curve from a representative experiment showing the effect of VPA on mean-squared displacement (<*d2*>). At the indicated time-point, VPA was added to the cell culture to a final concentration of 3 mM. Control (open circle) and VPA-treated (solid circle) cells were recorded at 2 and 1 min intervals, respectively. (c-f) Effects of VPA on the degree of Erk1/2 phosphorylation in L929 cells. (c, e) Representative immunoblots showing the degree of Erk1/2 phosphorylation in cells grown in the presence or absence of 3 mM VPA for 20 min to 24 h (c) or the presence or absence of 0-3 mM VPA for 24 h (e). The corresponding levels of Erk1/2 are shown as loading controls. (d, f) Time- and dose-response graphs showing the effects of 3 mM VPA for 0-24 h (d) or 0-3 mM VPA for 24 h (f) on the degree of Erk1/2 phosphorylation, which was normalized to the corresponding cells untreated with VPA from four to five individual experiments.

**Figure **3a shows time-dependent effects of 3 mM VPA on the mean-cell speed of L929 cells. The speed was significantly reduced after 1 h exposure to the drug. After 24-48 h exposure, further reductions in cell speed were observed. **Figure **3b shows data from the time-response experiments, in which VPA was directly added to the culture medium during the motility recordings. Using this approach, a reduction in cell displacement was observed for VPA-treated cells ~20 min after drug addition compared with control cultures. These observations demonstrate that VPA caused biphasic, time-related inhibition of the cell speed of L929 cells, characterized by an initial, rapid inhibition followed by a further inhibition at later time-points.

**Figure **3c and 3e show time-response effects of VPA on the degree of Erk1/2 phosphorylation. A 1 h exposure to 3 mM VPA significantly decreased the degree of Erk1/2 phosphorylation. After 6 and 24 h, the degree of Erk1/2 phosphorylation decreased further. **Figure **3d and 3f show dose-response curves of the effects of VPA on Erk1/2 phosphorylation. VPA dose-dependently inhibited the degree of Erk1/2 phosphorylation, with estimated IC_25 _and IC_50 _values of ~0.24 and 0.58 mM, respectively. A comparison of **Figure **3a and 3d revealed that the time-course of the effects of VPA on the degree of Erk1/2 phosphorylation resembled that observed for the inhibition of cell speed, suggesting that VPA partially modulated cell speed through modulation of the degree of Erk1/2 phosphorylation.

**Figure 4:**
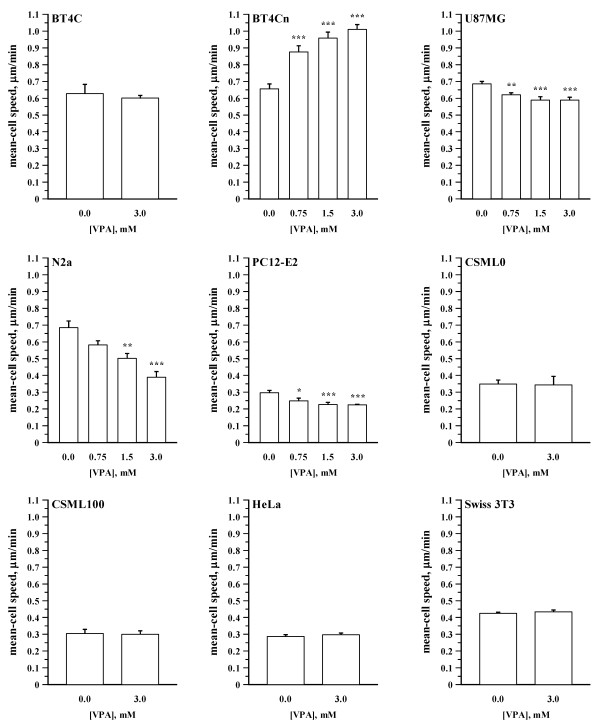
**Effects of VPA on individual cell motility**. Bar graphs showing the effects of VPA on the cell speed. Cells were exposed to VPA (0-3 mM, 48 h) before individual cell motility was recorded. BT4C and BT4Cn cells were recorded for 20 min at 5 min intervals. The remaining cell lines were recorded for 40 min at 10 min intervals. Results were normalized to the corresponding cells untreated with VPA from four to six independent experiments, each being based on analysis 50-200 cells/treatment.

Following the studies of L929 cells, the motility of the remaining nine cell lines was investigated. **Figure **4 shows that VPA significantly and dose-dependently inhibited the speed of N2a, U87MG and PC12-E2 cells, stimulated the speed of BT4Cn cells, and did not affect the speed of the remaining five cell lines (BT4C, CSML0, CSML100, HeLa and Swiss 3T3).

### Effects of VPA on individual cell motility by modulation of signal transduction upstream of Erk1/2

**Figure 5:**
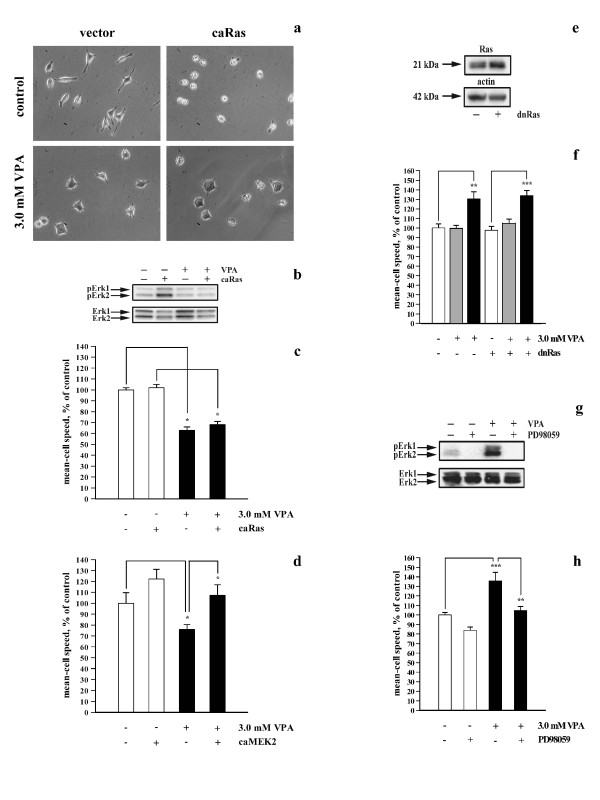
**Effects of VPA on individual cell motility by modulation of signal transduction upstream of Erk1/2**. (a) Micrographs of L929 cells stably expressing or not expressing constitutively active Ras (caRas) and grown in the absence or presence of VPA (3 mM, 48 h). (b) Representative immunoblot showing the degree of Erk1/2 phosphorylation in cells grown in the presence or absence of VPA (3 mM, 48 h). (c) Bar graph showing the mean-cell speed of cells grown in the presence or absence of VPA (3 mM, 48 h). (d) Bar graph showing the mean-cell speed of L929 cells transiently expressing or not expressing constitutively active MEK2 (caMEK2). Cells grown in the presence or absence of VPA (3 mM, 4 h) were recorded 48 h after transfection. Data presented in c and d are from cells recorded for 50 min at 10 min intervals. Results were normalized to the corresponding controls from four individual experiments. (e) Representative immunoblot showing the expression of Ras in BT4Cn cells transiently expressing or not expressing dominant-negative Ras (dnRas). Lysates were prepared 48 h after transfection. (f) Bar graph showing the mean-cell speed of BT4Cn cells grown in the presence or absence of VPA (3 mM, 4 or 24 h) and recorded for 20 min at 2 min intervals (48 h after transfection). Results were normalized to the corresponding controls from 6 individual experiments. (g) Representative immunoblot showing the degree of Erk1/2 phosphorylation in BT4Cn cells grown in the presence or absence of VPA (3 mM, 48 h) and treated or untreated with 25 μM PD98059 for 1 h. (h) Bar graph showing the mean-cell speed of BT4Cn cells grown in the presence or absence of VPA (3 mM, 48 h) and treated or untreated with 25 μM PD98059 for 1 h. Recordings were performed for 20 min at 5 min intervals. Results were normalized to the corresponding controls from eight individual experiments.

The GTPase Ras is an upstream activator of the MAPK pathway [11]. To determine whether VPA modulated the MAPK pathway upstream or downstream of Ras, the effects of VPA were investigated in L929 cells expressing constitutively active Ras (caRas). **Figure **5a shows micrographs of L929 cells not expressing or expressing caRas and grown in the presence or absence of VPA. Control-transfected cells adopted a stellate phenotype with an increased area when treated with VPA (**Figure **5a, two leftmost images), whereas caRas-expressing cells were more round and loosely attached (**Figure **5a, two topmost images), a typical phenotype of Ras-transformed cells [22]. However, exposure of caRas-expressing cells to VPA caused them to adopt a phenotype similar to control-transfected cells treated with VPA (**Figure **5a, two rightmost images). The immunoblot presented in **Figure **5b shows that L929 cells expressing caRas exhibited higher degrees of Erk1/2 phosphorylation than control-transfected cells when grown in the absence of VPA (**Figure **5b, lanes 2 and 1). However, VPA caused equally strong inhibition of Erk1/2 phosphorylation in control- and caRas-transfected cells (**Figure **5b, lanes 3 and 4). These results demonstrate that exposure to VPA reversed the effects of caRas expression on L929 cell morphology and the degree of Erk1/2 phosphorylation. Cells expressing caRas did not move significantly faster than control-transfected cells when grown in the absence of VPA (**Figure **5c). The speed of control- and caRas-transfected cells was similarly inhibited in the presence of VPA (**Figure **5c).

**Figure **5d shows the effects of VPA exposure on the speed of L929 cells expressing constitutively active MEK2 (caMEK2). Cells expressing caMEK2 did not move significantly faster than control-transfected cells grown in the absence of VPA (**Figure **5d, p < 0.063). However, in the presence of VPA, caMEK2-expressing cells moved significantly faster than control-transfected cells exposed to VPA (**Figure **5d, p < 0.029). Moreover, the speed of caMEK2-expressing cells was not significantly reduced after exposure to VPA (**Figure **5d, column 4 vs. column 2).

To determine whether VPA affected the speed of BT4Cn cells through effects on the degree of Erk1/2 phosphorylation, the effects of VPA were investigated in BT4Cn cells expressing dominant-negative Ras (dnRas). The expression of dnRas was verified by immunoblotting using a polyclonal anti-Ras antibody. As shown in **Figure **5e, Ras immunoreactivity was increased in cells transfected with the dnRas expression vector, indicating that the cells expressed dnRas. **Figure **5f shows that exposure of BT4Cn cells to 3 mM VPA for 24 h, but not 4 h, significantly increased the speed of both control- and dnRas-transfected cells. Thus, the expression of dnRas was not able to prevent the VPA-induced increase in the speed of BT4Cn cells.

**Figure **5g shows an immunoblot of BT4Cn cells treated or untreated with VPA and/or the MEK inhibitor PD98059. As expected, VPA increased the degree of Erk1/2 phosphorylation compared with untreated cells (**Figure **5g, lanes 1 and 3). However, this stimulation was prevented by treating the cells with PD98059 (**Figure **5g, lane 4). Similarly, treatment of BT4Cn cells with VPA increased the cell speed compared with untreated cells (**Figure **5h, columns 1 and 3). However, the VPA-induced increase of the cell speed was completely prevented by PD98059 (**Figure **5h, column 3 vs. column 4), which reduced the speed of VPA-treated cells to the level of control cells untreated with VPA and PD98059 (**Figure **5h, columns 1 and 4). The same concentration of PD98059 did not significantly reduce the speed of cells untreated with VPA (**Figure **5h, columns 1 and 2).

In conclusion, the data presented in **Figure **5a-h demonstrate that VPA in L929 and BT4Cn cells modulates cell motility, cell morphology and the degree of Erk1/2 phosphorylation by altering signaling through the MAPK pathway downstream of Ras but upstream of MEK (i.e., at the level of Raf).

## Discussion

The observation that VPA is an HDAC inhibitor [6,23] has spurred numerous studies demonstrating that VPA possesses anti-cancer properties *in vitro *and *in vivo *[2,5,24]. However, VPA affects the activities of several enzymes and signal transduction pathways, and the mechanisms underlying the anti-cancer properties of VPA are not well characterized.

Consistent with earlier studies [25,26], we demonstrated that the degree of VPA-induced histone H3 acetylation was highly cell type-specific. Moreover, we found that the effect of VPA on the degree of Erk1/2 phosphorylation was highly cell type-specific. This observation is in contrast to the general notion that VPA, as demonstrated in several studies [17,27-30], activates Erk1/2, although inhibition has also been reported [16].

HDAC inhibitors can inhibit Erk1/2 activity [31,32]. However, consistent with recent studies [29,33], no relationship was observed between HDAC inhibition and the corresponding changes in the degree of Erk1/2 phosphorylation (**Additional file **1, **Figure S4**). Therefore, effects of VPA on the degree of Erk1/2 phosphorylation and HDAC inhibition seem to be independent responses that, subsequently, may modulate biological processes such as cell growth or motility through independent mechanisms.

VPA-induced HDAC inhibition can hypothetically affect cell motility. Thus, VPA has in some [34], but not all [35], studies been shown to inhibit HDAC6, an enzyme known to modulate cell motility [36]. Likewise, VPA-induced HDAC inhibition can hypothetically affect cell growth. However, we did not find any correlations between the effects of VPA on HDAC activity and cell motility or growth (**Additional file **1, **Figure S4**).

Erk1/2 activity controls multiple processes, including cell cycle progression, and cell growth, motility and survival [37]. Therefore, VPA-induced changes in Erk1/2 activity can hypothetically affect cell growth and motility. Hence, we focused our attention on the possible relationship between VPA-induced changes in Erk1/2 activity, cell growth and motility.

Cell growth can be inhibited by both a sustained increase and decrease in Erk1/2 activity [37]. Therefore, no general correlation was found between the effects of VPA on the degree of Erk1/2 phosphorylation and cell growth (**Additional file **1, **Figure S4**). However, cell lines demonstrating significant changes in the degree of Erk1/2 phosphorylation in response to VPA, generally had lower IC_50 _values for growth than cell lines with unaffected degrees of Erk1/2 phosphorylation (pERK-change, p < 0.05 and growth IC_50 _>3 mM, 0 cell lines; pERK-change, p < 0.05 and growth IC_50 _<3 mM, 5 cell lines; pERK-change, p > 0.05 and growth IC_50 _<3 mM, 4 cell lines; pERK-change, p > 0.05 and growth IC_50 _>3 mM, 1 cell line; p < 0.05; χ^2 ^and Fisher's exact test). Therefore, we hypothesize that the modulation of the degree of Erk1/2 phosphorylation by VPA is of central importance for drug-mediated inhibition of cell inhibition.

We originally demonstrated VPA to inhibit the cell speed [21]. However, consistent with later studies [15,38,39], the present study shows that the effects of VPA on the cell speed are highly cell type specific. Interestingly, a time-response of the VPA-induced change in L929 mean-cell speed exhibited a biphasic response, with a significant reduction detectable already after ~20 min followed by a further decrease after 24-48 h. Hence, the initial, rapid response must be independent of alterations in gene transcription, whereas the changes at later time-points may be the result of alterations in gene transcription.

The Ras-MAPK pathway regulates cell motility both independent of, and as a result of, changes in gene transcription [8]. However, Ras-MAPK signaling can affect different cell types differently. For example, VPA increased the degree of Erk1/2 phosphorylation in BT4Cn and N2a cells. However, BT4Cn cells maintained a de-differentiated phenotype, and exhibited an increase in both lamellipodia (**Additional file **1, **Figure S2 **and **Table S1**) and the cell speed, whereas N2a cells, known to differentiate in response to a sustained increase in Erk1/2 activity [40], consequently demonstrated a decrease in the cell speed when exposed to VPA. Therefore, a direct correlation between changes in the degree of Erk1/2 phosphorylation and the cell speed is not to be expected and was not observed (**Additional file **1, **Figure S4**). Nevertheless, a relationship was found since both L929 and BT4Cn cells demonstrated opposite effects with respect to changes in the degree of Erk1/2 phosphorylation and cell speed in response to VPA. Moreover, in both cell lines the effect of the drug on the Ras-MAPK pathway could be observed at a position downstream of Ras but upstream of MEK (i.e., at the level of Raf). This observation is consistent with a previous study in which abrogation of Ras signaling by preventing the farnisylation of the protein did not affect VPA-mediated activation of Erk1/2 in endothelial cells [29].

Raf exists in three isoforms, A-, B- and c-Raf [13], which respond differently to Ras-independent upstream activators. PKA can stimulate the activity of B-Raf but inhibits the activity of c-Raf [41], which instead can be activated by PKC [14]. Consequently, cell type-specific effects of VPA on the degree of Erk1/2 phosphorylation may be partially explained by cell type-specific differences in the expression of Raf isoforms. An analysis of Raf expression revealed that all three Raf isoforms were expressed in all 10 investigated cell lines, although at highly variable levels (**Additional file **1, **Figure S3**). However, no apparent relationship was found between the expression of the respective Raf isoforms and the observed changes in the degree of Erk1/2 phosphorylation in response to VPA. Notably, constitutively activated B- and c-Raf mutations are frequently observed in human cancers [42]. However, the analysis of Raf expression did not include studies of Raf mutations, and therefore the possibility that the expression of mutated Raf isoforms can contribute to the observed results cannot be excluded.

VPA has been suggested to be a possible chemotherapeutic drug for the treatment of gliomas [4,5]. However, consistent with recent observations [43], we found an increase in the growth of the human glioma U87MG at a physiologically relevant concentration of VPA. Moreover, the cell speed of the malignant glioma BT4Cn was profoundly increased in response to VPA. These observations suggest that VPA should be used with caution for the treatment of gliomas.

## Conclusions

In conclusion, we demonstrate that VPA exposure induced considerable cell type-specific effects on HDAC inhibition, Erk1/2 phosphorylation, cell growth and motility. Furthermore, Erk1/2 phosphorylation, cell growth and motility are modulated independently of the degree of HDAC inhibition. In contrast, VPA affects signaling through the MAPK pathway at the level of Raf, thereby modulating cell growth and motility. These results suggest that the cell type-specific effects of VPA on the activity of Erk1/2 are important in relation to the use of VPA as an anti-cancer drug.

## List Of Abbreviations

caMEK2: constitutively active MEK2; caRas: constitutively active Ras; DMEM: Dulbecco's modified Eagle's medium; DMSO: dimethylsulfoxide; dnRas: dominant negative Ras; FCS: fetal calf serum; GSK-3β: glycogen synthase kinase-3β; HDAC: histone deacetylase; MAPK: mitogen-activated protein kinase; PKA: protein kinase A; PKC: protein kinase C; SDS-PAGE: sodium dodecyl sulfate-polyacrylamide gel electrophoresis; VPA: valproic acid.

## Competing interests

The authors declare that they have no competing interests.

## Authors' contributions

EAL made the Ras-transfected cell lines. KG, GS and PSW made all experiments. KG, GS, VB, EB, and PSW participated in the study design. PSW and KG wrote the manuscript. All authors read and approved the final manuscript.

## Pre-publication history

The pre-publication history for this paper can be accessed here:

http://www.biomedcentral.com/1471-2407/10/383/prepub

## Supplementary Material

Additional file 1 **Supplementary methods**. Supplementary references. Supplementary Table 1: Effects of VPA on cell morphology. Supplementary figure legends. Supplementary figure 1: Effects of VPA on individual cell motility. (9 graphs) Supplementary figure 2: Effects of VPA on cell morphology. (20 micrographs) Supplementary figure 3: Analysis of A-, B-, and c-Raf expression. (3 agarose gels, 4 western blots) Supplementary figure 4: Relationships between changes in the degree of Erk1/2 phosphorylation, cell growth, cell speed, and histone H3 acetylation in response to VPA treatment. (5 graphs) Click here for file
