# Supplementary material for: Cell type-specific anti-cancer properties of valproic acid: independent effects on HDAC activity and Erk1/2 phosphorylation
Source: BMC Cancer. 2010 Jul 21;10:383. doi: 10.1186/1471-2407-10-383 (PMC2918577; doi:10.1186/1471-2407-10-383)
Supplement: Additional file 1 — Supplementary methods. Supplementary references. Supplementary Table 1: Effects of VPA on cell morphology. Supplementary figure legends. Supplementary figure 1: Effects of VPA on individual cell motility. (9 graphs) Supplementary figure 2: Effects of VPA on cell morphology. (20 micrographs) Supplementary figure 3: Analysis of A-, B-, and c-Raf expression. (3 agarose gels, 4 western blots) Supplementary figure 4: Relationships between changes in the degree of Erk1/2 phosphorylation, cell growth, cell speed, and histone H3 acetylation in response to VPA treatment. (5 graphs) [file 1471-2407-10-383-S1.PDF]

SUPPLEMENTARY MATERIAL

SUPPLEMENTARY METHODS

Immunoblotting

Immunoblotting was performed as described in the Materials and Methods of the main article using antibodies against A-Raf (Cell Signaling), B-Raf, and c-Raf (C-19 and C-12, respectively; both from Santa Cruz Biotechnology, Heidelberg, Germany). For the normalization of the degree of A-, B-, and c-Raf immunoreactivity, membranes were stripped and reprobed with anti-actin antibody (Sigma-Aldrich, Copenhagen, Denmark).

Raf mRNA expression profiling

Total RNA pools from subconfluent cell cultures were isolated using RNeasy Mini Kit (Qiagen, Solna, Sweden) according to the manufacturer's recommendations. Subsequently, reverse transcription polymerase chain reaction (RT-PCR) was performed using DNase I-digested (Invitrogen, Taastrup, Denmark) total RNA (~500 ng RNA/reaction) essentially as previously described [1]. The following primers were used: A-Raf sense, 5' – GCA GTG GGC ACC GTC AAA GTA TAC – 3'; A-Raf antisense, 5' – GCA ATG GCT GTG TCC CAG G – 3' (Entrez Nucleotide accession no. NM_001654, product length 188 bp); B-Raf sense, 5' – CAG GAA CAT ATA GAG GCC CTA TTG GAC – 3', B-Raf antisense, 5' – CCA GTA AGC CAG GAA ATA TCA GTG TCC C – 3' (Entrez Nucleotide accession no. NM_004333, product length 485 bp); c-Raf sense, 5' – AGC TGC ATC TCT CCT ACA ATA GTT CAG C – 3'; c-Raf antisense, 5' – GGT GAA GGC GTG AGG TGT AGA ATA TCT G – 3' (Entrez Nucleotide accession no. NM_002880, product length 642 bp). Polymerase chain reactions (PCR) were run for 35 cycles using an annealing temperature of 55ºC. PCR products were electrophoresed on 1% (w/v) agarose gels containing ethidium bromide. The experiments were performed using two independent total

RNA samples from each cell line. The identities of the obtained PCR products were verified by sequencing (GATC Biotech, Konstanz, Germany).

Fluorescence microscopy

Cells were plated in 4-well Permanox LabTek chamber slides (Nunc, Roskilde, Denmark) at a density of ~2 × 10^3 cells/well and grown in the presence or absence of VPA (3 mM, 48 h). Subsequently, cells were rinsed in actin-stabilizing buffer (ASB; 10 mM Tris, 0.15 M NaCl, pH 7.4, 2 mM $MgCl_2$, and 10% glycerol (v/v) [2]), fixed in 4% (w/v) formaldehyde in ASB, permeabilized with 0.2% (w/v) saponin in ASB, and stained using Alexa 488-conjugated phalloidin (Invitrogen) for the visualization of F-actin. Subsequently, cells were incubated with Sytox Orange (Invitrogen) for the visualization of cell nuclei. The specimens were mounted using Prolong Gold Antifade Reagent (Invitrogen). Confocal micrographs were obtained using a Radiance 2000 confocal laser scanning system (BioRad Laboratories, Copenhagen, Denmark) attached to an inverted Nikon Eclipse TE 200 microscope equipped with a 60× objective.

SUPPLEMENTARY REFERENCES:

1. Neiiendam JL, Kohler LB, Christensen C, Li S, Pedersen MV, Ditlevsen DK, Kornum MK, Kiselyov VV, Berezin V, Bock E: **An NCAM-derived FGF-receptor agonist, the FGL-peptide, induces neurite outgrowth and neuronal survival in primary rat neurons**. *Journal of Neurochemistry* 2004, **91**:920-935.
2. Knowles GC, McCulloch CA: **Simultaneous localization and quantification of relative G and F actin content: optimization of fluorescence labeling methods**. *J Histochem Cytochem* 1992, **40**:1605-1612.

FIGURE LEGENDS:

Fig. S1: Effects of VPA on individual cell motility.

The graphs show the same data as Fig. 4. The dose-response curves show mean-squared displacement ($<d^2>$) of the investigated cell lines grown in the absence (black lines) or presence of 0.75, 1.5, and 3 mM VPA (blue, grey, and red lines, respectively). Results are expressed as mean ± SEM on the basis of four to six independent experiments. The curves confirm that cells, which did not exhibit altered motility in response to VPA, were not non-motile.

Fig. S2: Effects of VPA on cell morphology.

Confocal micrographs showing the morphology of cells grown in the presence or absence of VPA (3 mM, 48 h). Cells were stained with Alexa 488-conjugated phalloidin for detection of F-actin (red) and Sytox Orange for detection of nuclei (green). The individual images show a composite of representative cells collected from different regions of a single microscope slide. Scale bar = 25 μm.

Fig. S3: Analysis of A-, B-, and c-Raf expression.

Expression of A-, B-, and c-Raf. (a) Representative qualitative RT-PCR analysis of mRNA expression of A-, B-, and c-Raf performed on total RNA. Arrows indicate the sizes (bp) of PCR products according to DNA ladder marker. (b) Representative semiquantitative immunoblots of protein expression of A-, B-, and c-Raf performed on total cell lysates. The corresponding actin levels are shown as loading controls. Arrows indicate the sizes (kDa) of protein bands according to protein marker.

Fig. S4: Relationships between changes in Erk1/2 activity, cell growth, cell speed, and histone H3 acetylation in response to VPA treatment.

The figures show the relationships between the relative changes in responses to VPA (3 mM, 48 h) with regard to the degree of Erk1/2 phosphorylation, cell growth, mean cell speed, and histone H3 acetylation. □, BT4C; ○, BT4Cn; △, U87MG; ▽, N2a; ✯, PC12-E2; ■, CSML0; ●, CSML100; ▲, HeLa; ▼, Swiss 3T3; ★, L929.

Table S1: Effects of VPA on cell morphology.

**Cell Line**	**Changes in Morphology**	
	Semiquantitative Changes	*Qualitative Changes*
BT4C	-	
BT4Cn	++	Larger area; more and bigger lamellipodia
U87MG	+	Larger area; more stress-fibers
N2a	+	More elongated
PC12-E2	+	More aggregated
CSML0	+	Smaller area
CSML100	+	Increased area; more elongated
HeLa	–	
Swiss 3T3	–	
L929	++	Larger area; more stress-fibers

Changes in cell morphology after exposure to VPA (3 mM, 48 h) were subjectively ranked based on visual inspection of fluorescence micrographs. (–) Lack of apparent changes in morphology; (+) moderate changes; (++) strong changes.

Fig. S1

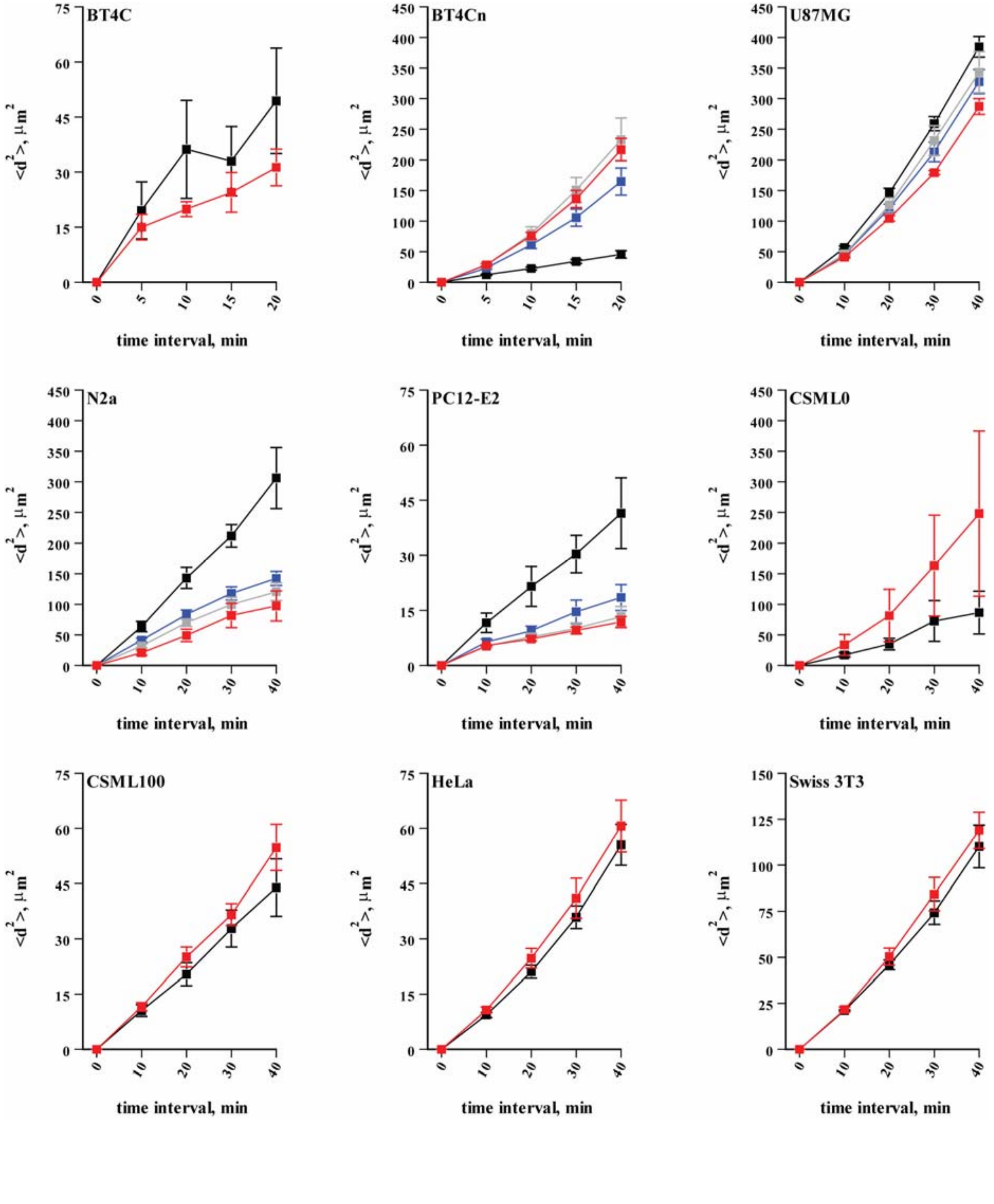
BT4C
BT4Cn
U87MG
N2a
PC12-E2
CSML0
CSML100
HeLa
Swiss 3T3
$<d^2>$, μm^2
time interval, min

Fig. S2

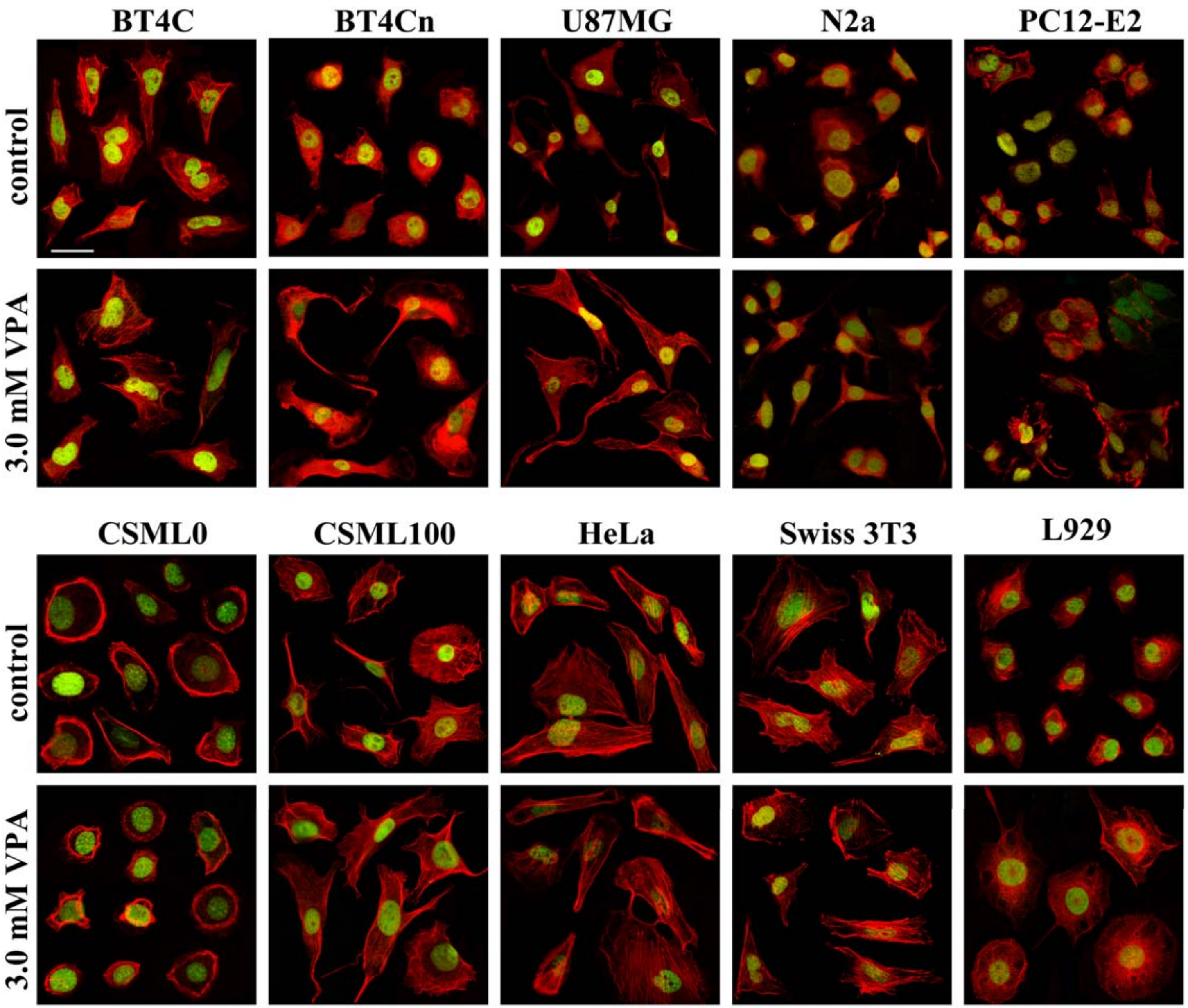
BT4C
BT4Cn
U87MG
N2a
PC12-E2
control
3.0 mM VPA
CSML0
CSML100
HeLa
Swiss 3T3
L929
control
3.0 mM VPA

Fig. S3

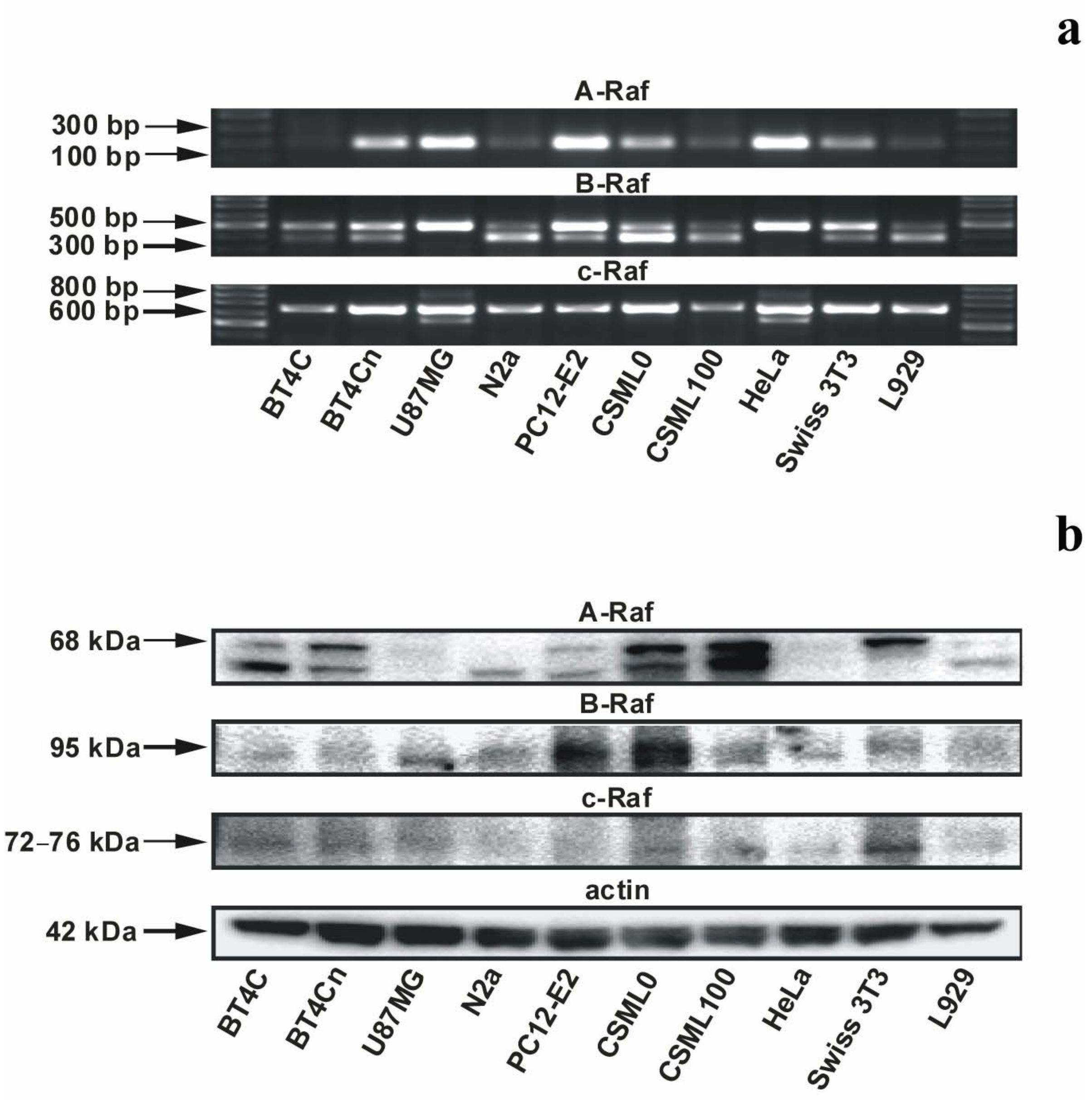
a
A-Raf
300 bp
100 bp
B-Raf
500 bp
300 bp
c-Raf
800 bp
600 bp
BT4C
BT4Cn
U87MG
N2a
PC12-E2
CSML0
CSML100
HeLa
Swiss 3T3
L929
b
A-Raf
68 kDa
B-Raf
95 kDa
c-Raf
72–76 kDa
actin
42 kDa
BT4C
BT4Cn
U87MG
N2a
PC12-E2
CSML0
CSML100
HeLa
Swiss 3T3
L929

Fig. S4

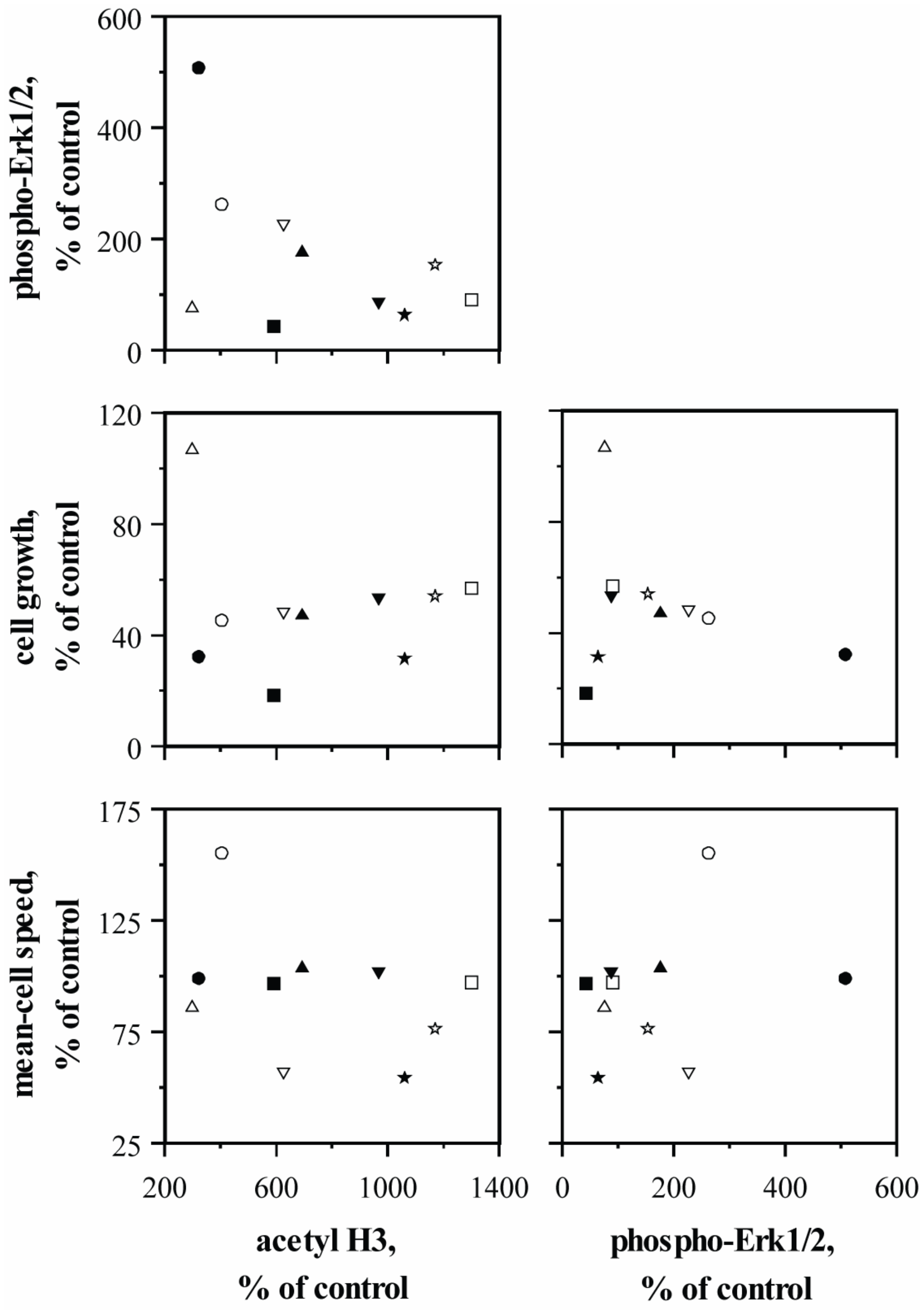

600
400
200
0
phospho-Erk1/2,
% of control
120
80
40
0
cell growth,
% of control
175
125
75
25
mean-cell speed,
% of control
200
600
1000
1400
0
200
400
600
acetyl H3,
% of control
phospho-Erk1/2,
% of control
